# Immersive Virtual Environments and Wearable Haptic Devices in rehabilitation of children with neuromotor impairments: a single-blind randomized controlled crossover pilot study

**DOI:** 10.1186/s12984-020-00771-6

**Published:** 2020-10-28

**Authors:** Ilaria Bortone, Michele Barsotti, Daniele Leonardis, Alessandra Crecchi, Alessandra Tozzini, Luca Bonfiglio, Antonio Frisoli

**Affiliations:** 1 Institute of Clinical Physiology (IFC), National Research Council (CNR), Pisa, Italy; 2 PERCRO Laboratory, Scuola Superiore Sant’Anna, Pisa, Italy; 3 Unit of Developmental Neurorehabilitation, Maternal and Child Department, Pisa University Hospital, Pisa, Italy

**Keywords:** Virtual reality, Human Motion Analysis, Rehabilitation, Tactile Feedback, Serious game

## Abstract

**Background:**

The past decade has seen the emergence of rehabilitation treatments using virtual reality. One of the advantages in using this technology is the potential to create positive motivation, by means of engaging environments and tasks shaped in the form of serious games. The aim of this study is to determine the efficacy of immersive Virtual Environments and weaRable hAptic devices (VERA) for rehabilitation of upper limb in children with Cerebral Palsy (CP) and Developmental Dyspraxia (DD).

**Methods:**

A two period cross-over design was adopted for determining the differences between the proposed therapy and a conventional treatment. Eight children were randomized into two groups: one group received the VERA treatment in the first period and the manual therapy in the second period, and viceversa for the other group. Children were assessed at the beginning and the end of each period through both the Nine Hole Peg Test (9-HPT, primary outcome) and Kinesiological Measurements obtained during the performing of similar tasks in a real setting scenario (secondary outcomes).

**Results:**

All subjects, not depending from which group they come from, significantly improved in both the performance of the 9-HPT and in the parameters of the kinesiological measurements (movement error and smoothness). No statistically significant differences have been found between the two groups.

**Conclusions:**

These findings suggest that immersive VE and wearable haptic devices is a viable alternative to conventional therapy for improving upper extremity function in children with neuromotor impairments.

*Trial registration* ClinicalTrials, NCT03353623. Registered 27 November 2017-Retrospectively registered, https://clinicaltrials.gov/ct2/show/NCT03353623

## Background

Neuromotor impairments in children show a great variability in both causes and clinical signs and they occur in a challenging developmental context. Treatment options are broad ranging, however effective therapeutic options for these children frequently remain an unmet need [1, 2]. New digital technologies offer an exciting means of engaging children in therapy and providing intervention environments and experiences that can easily be adapted to individual needs [3]. Since it is well known that playing is an innate activity that enables humans to learn and grow, the research community and the game industry have moved towards the development of serious games (SG), incorporating both pedagogical and entertaining elements. However, there is the need to standardize procedures and to translate them into clinical practice in order to assess these therapeutic strategies in appropriately well designed pilot trials [4, 5].

**Table 1:** Main clinical and technological features in literature review of VR-assisted rehabilitation for Upper Limb Therapy in children with neuromotor impairments

References	Participants (design)	Intensity	Intervention	Technology	Achievements
Chiu et al. [6]	62 CP (AB)	6 ses	VR vs CONV	Commercially available	No effects
Hammond et al. [7]	28 DCD (AB)	12 ses	VR vs CONV	Commercially available	No effects
Zoccolillo et al. [8]	22 CP (AB)	16 ses	VR vs CONV	Commercially available	Therapy effects
Shin et al. [9]	16 CP (AB)	16 ses	VR vs CONV	Commercially available	Period effect
Gilleaux et al. [10]	16 CP (AB)	40 ses	VR + PT + OT vs PT + OT	Laboratory	Period $\times$ Group effect
Howie et al. [11]	21 DCD (ABBA)	16 weeks	VR - CONV vs CONV - VR	Commercially available	No effects
Preston et al. [12]	15 CP (AB)	6 weeks	VR + CONV vs CONV	Laboratory	No effects
Sakzewski et al. [13]	58 ABI (AB)	120 ses	VR vs CONV	Commercially available	No effects
Bedair et al. [14]	40 CP (AB)	96 ses	VR + PT vs CONV	Commercially available	Therapy effects
Sajan et al. [15]	20 CP (AB)	18 ses	VR vs CONV	Commercially available	Therapy effects
Kassee et al. [16]	6 CP (AB)	6 weeks	VR vs PT	Commercially available	No effects
El Shamy [17]	30 CP (AB)	36 ses	VR vs CONV	Laboratory	Therapy effects
Cavalcante Neto et al. [18]	32 DCD	8 ses	VR vs CONV	Commercially available	Therapy effects
Tarakci et al. [19]	30 CP + 43 JIA + 19 BPBI (AB)	24 ses	LMC - VR vs CONV	Commercially available	Period effects

We reviewed all research studies published over the last 5 years, where VR-based interventions were promoted for upper limb therapy and have been conducted with children with neuromotor impairments (cerebral palsy, CP, or developmental dyspraxia, DD). We used Scopus and Isi Web of Knowledge electronic databases, based on the following keywords: (“virtual reality” OR “serious game” OR “virtual environment” OR “videogame” OR “robot-assisted”) AND (“rehabilitation” OR “therapy” OR “treament” OR “intervention”) AND (“upper limb” OR “upper extremity”) AND (“cerebral palsy” OR “cp” OR “dyspraxia” OR “developmental coordination disorder” OR “dcd ) AND (“child” OR “children”). We excluded reviews, trials with no pre/post comparison, theses or dissertations, and articles not published in the English language. Our initial search yielded 97 citations of journal articles written in English. After duplicates removal, we discarded 18 reviews and reviewed the remaining 45 studies in more detail. We excluded 31 of these studies because they did not meet all inclusion criteria. The final 14 eligible studies are described in Table 1.

From the literature analysis, it emerged a widespread interest in using VR-assisted rehabilitation in children with either CP or DD to address upper limb impairments. However, most of the study relied on intervention with low-cost, off-the-shelf VR system (Wii Sport Resort [6], Xbox360 with Microsoft Kinect [8], Playstation with Move and/or EyeToy [20]) without considering a specific clinical outcome for the patients.

Almost half of the studies reported no effects after the VR-assisted therapy. Chiu et al. [6] and Hammond et al. [7] showed that Wii training did not improve coordination, strength, or hand function. Also Howie et al. [11], Preston et al. [12] and Sakzewski et al. [13] showed the improvements did not reach minimal clinically important difference. Finally, Kassee et al. [16] reported only minimal improvements in the ABILHAND-Kids for all participants, with no differences among study groups. However, other studies showed significant improvements after the VR-session with regards to conventional treatments. Zoccolillo et al. [8] concluded that VR games (Xbox360 with Microsoft Kinect) resulted effective in improving motor functions, but failed in improving the manual abilities for performing activities of daily living which benefited most from the conventional therapy. Bedair et al. [14] investigated the effect of VR games (Xbox) as an adjunct treatment tool on upper extremity function in management of spastic hemiplegic children. They found significant differences in improvement between groups post-treatment in terms of visual motor skills. El-Shamy et al. [17] showed improvements after ARMEO robotic therapy, compared with conventional therapy, in children with hemiplegic CP, with Modified Ashworth Scale and the Quality of Upper Extremity Skills Test. Sajan et al. [15] and Cavalcante Neto et al. [18] concluded that task-specific training affords stronger benefits for general motor skill than Wii-based training. Only two studies reported no differences among the therapeutic approaches, thus suggesting that VR-assisted therapy was feasible as an alternative method for rehabilitation in children with neuromotor impairments [8, 19]. Further studies are then needed to verify whether VR-assisted training can promote clinically significant benefits for upper-limb (UL) function in children with neuromotor impairments and more standardized clinical trial should be encouraged.

The above mentioned studies, albeit exploiting motion capturing technologies for promoting motor recovery, did not provide kinesiological measurements for the improvements which are considered valuable clinical instrument for determining the effect of a rehabilitation therapy [21]. In order to increase patients’ motivation, which is a crucial factor for positively affecting the neuroplastic changes [22], immersion in VR can be further increased by involving multiple sensory pathways in the virtual experience. The addition of sense of touch has been considered since it is directly involved in a number of finalized motor tasks (e.g. pick and place). Wearable haptic devices, recently proposed in literature [23], are methods suited for delivering sense of touch in immersive virtual reality. Congruency of sense of touch with visual and auditory feedback has been proven to enhance sense of presence in the VR [24] and thus, we expect, to further involve the subject in the proposed virtual scenario.

In this work we present a randomized controlled crossover study comparing an immersive Virtual Environments and weaRable hAptic devices treatment (VERA) versus a conventional therapy. The proposed VERA treatment consists in an engaging multi-sensory serious game, already presented in [1, 2], designed with the aim to improve the movement of upper extremity of children with either CP or DD in both frontal and transverse plane. We hypothesized that the proposed therapy could be effective in improving functionality of the upper limb, measured through both functional test (nine hole peg test) and kinesiological assessment. The rest of the paper is organized as follows: firstly the methods are presented including the clinical trial design, the description of the therapies and the data analysis. Then, obtained results are reported and discussed in the last section.

## Methods

### Participants

Eight children (mean age = $10.13 \pm 2.59$ years old) were recruited from the Unit of Developmental Neurorehabilitation, Maternal and Child Department, Pisa University Hospital. All the patients were right-handed and they did not have previous experiences with VR and/or haptic devices. The inclusion criteria were a history of neuromotor impairments (cerebral palsy, CP, or developmental dyspraxia, DD), a maximum age of 18 years and low to severe impairments of the upper limbs (mantaining a minimum ability to actively grasp an object and the ability to understand simple instructions). The exclusion criteria were epileptic patients, severe deficit in sensory perception of upper limb, severe visual impairments and severe cognitive involvement. Children and parents provided written informed consent for participating in the study.

**Fig. 1:**
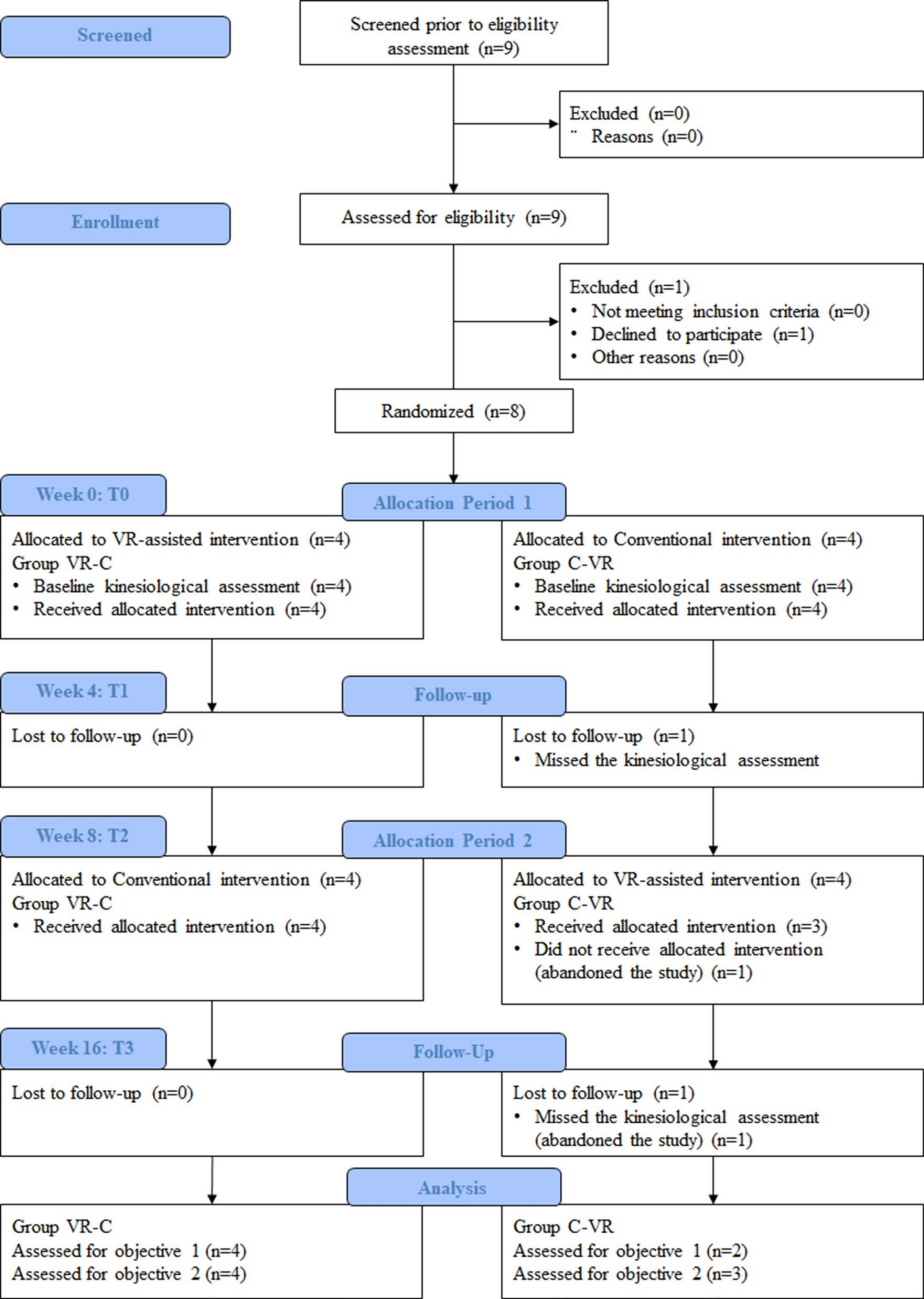
CONSORT flow diagram. CONSORT flow diagram illustrating participant flow during the different phases of the study. Flow of participants, withdrawals, and inclusion in analysis are described [45]

**Fig. 2:**
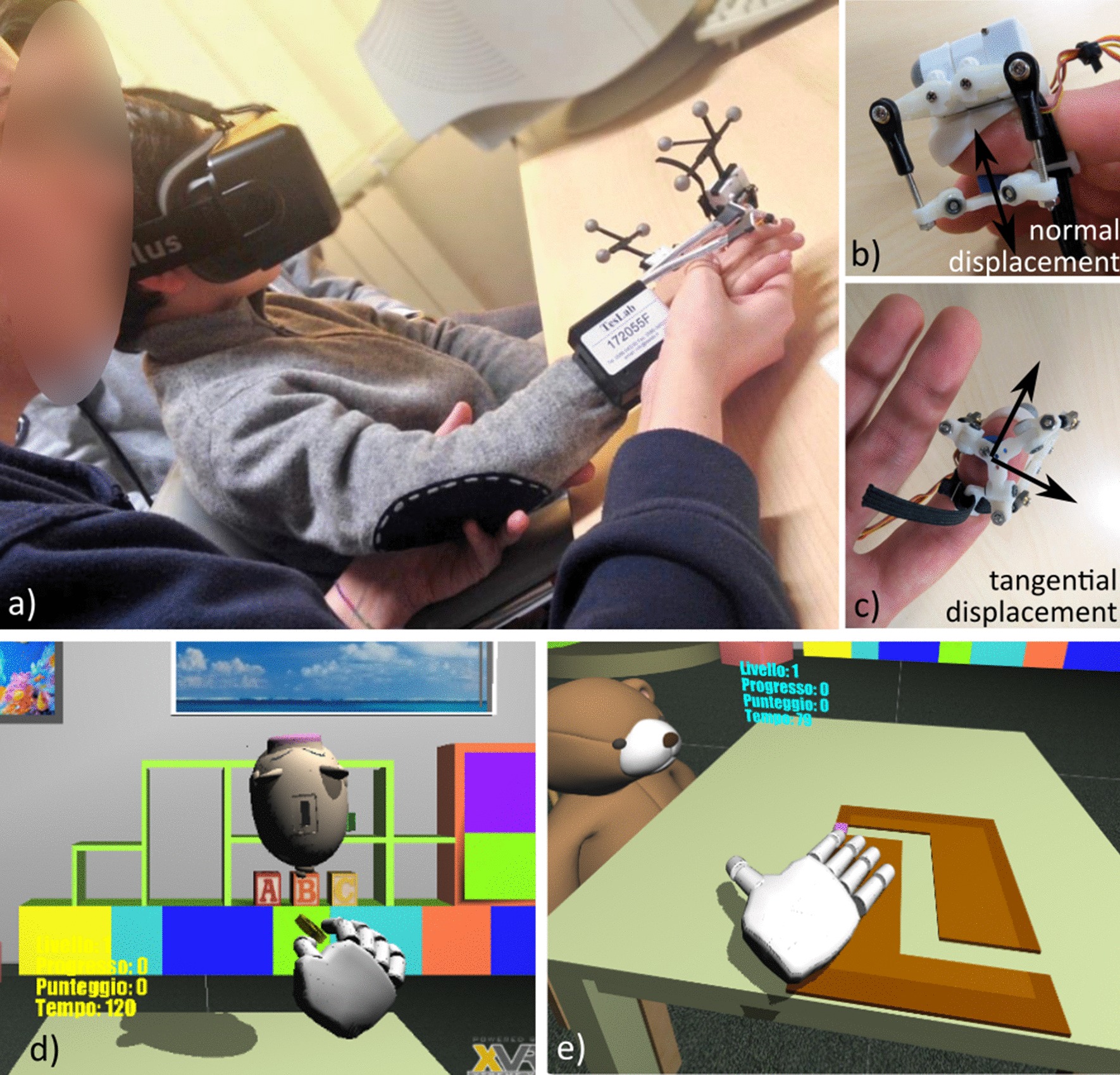
Set up illustration. **a** Screenshot of one of the experimental rehabilitation session with the VERA system. **b**, **c** Show detail of the fingertip haptic device rendering tangential and normal contact forces through cutaneous feedback [23, 46]. Screenshots of the virtual gaming scenarios [1, 2, 39] involving grasping and reaching task in the frontal plane (**d**), and path tracking tasks in the horizontal plane (**e**)

### Design

A two period cross-over design was adopted in this study for investigating if differences exist between the proposed approach and conventional therapy. The patients were equally randomized into 2 groups. One group underwent the VR-assisted intervention in the first period and the Conventional therapy in the second (VR-C group) while the other group underwent the Conventional therapy in the first period and the proposed therapy in the second (C-VR group).

Figure 1 provides an overview of the study according to the CONSORT statement. Children from the VR-C group received 8 hours (2 sessions per week for 4 weeks) of VERA rehabilitation before receiving the conventional/ongoing therapy. Children from the C-VR group received 8 hours (2 sessions per week for 4 weeks) of conventional/ongoing therapy before receiving the VERA rehabilitation. A wash-out period of 4 weeks was included between the two treatment period.

**Fig. 3:**
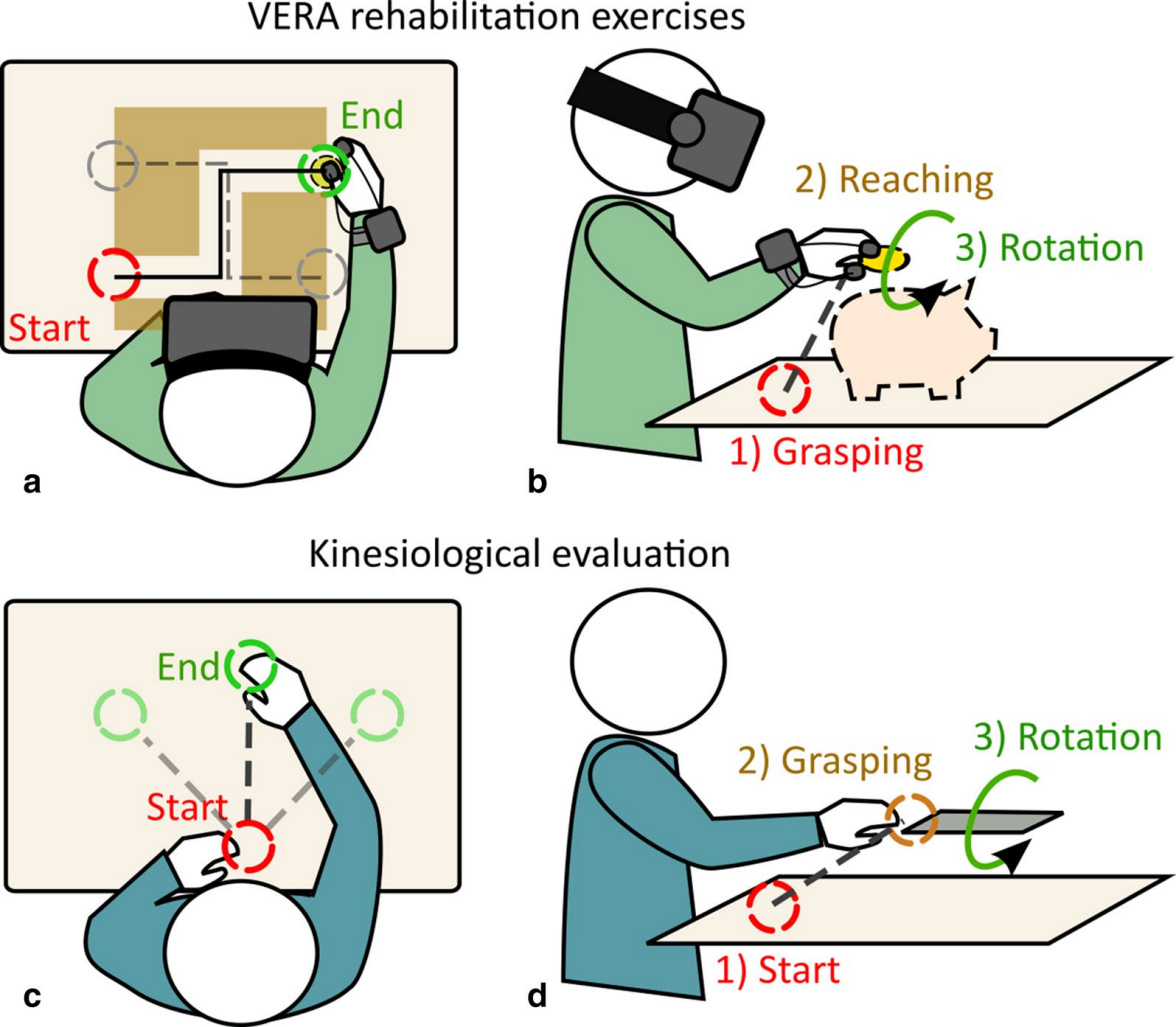
Layout of VERA Rehabilitation Therapy. Layout of the exercises proposed in the VERA treatment, involving planar tracking (**a**) and reach-to-grasp (**b**) motor tasks, and the layout of the similar tasks proposed in the kinesiological assessment (**c**, **d** respectively)

**Fig. 4:**
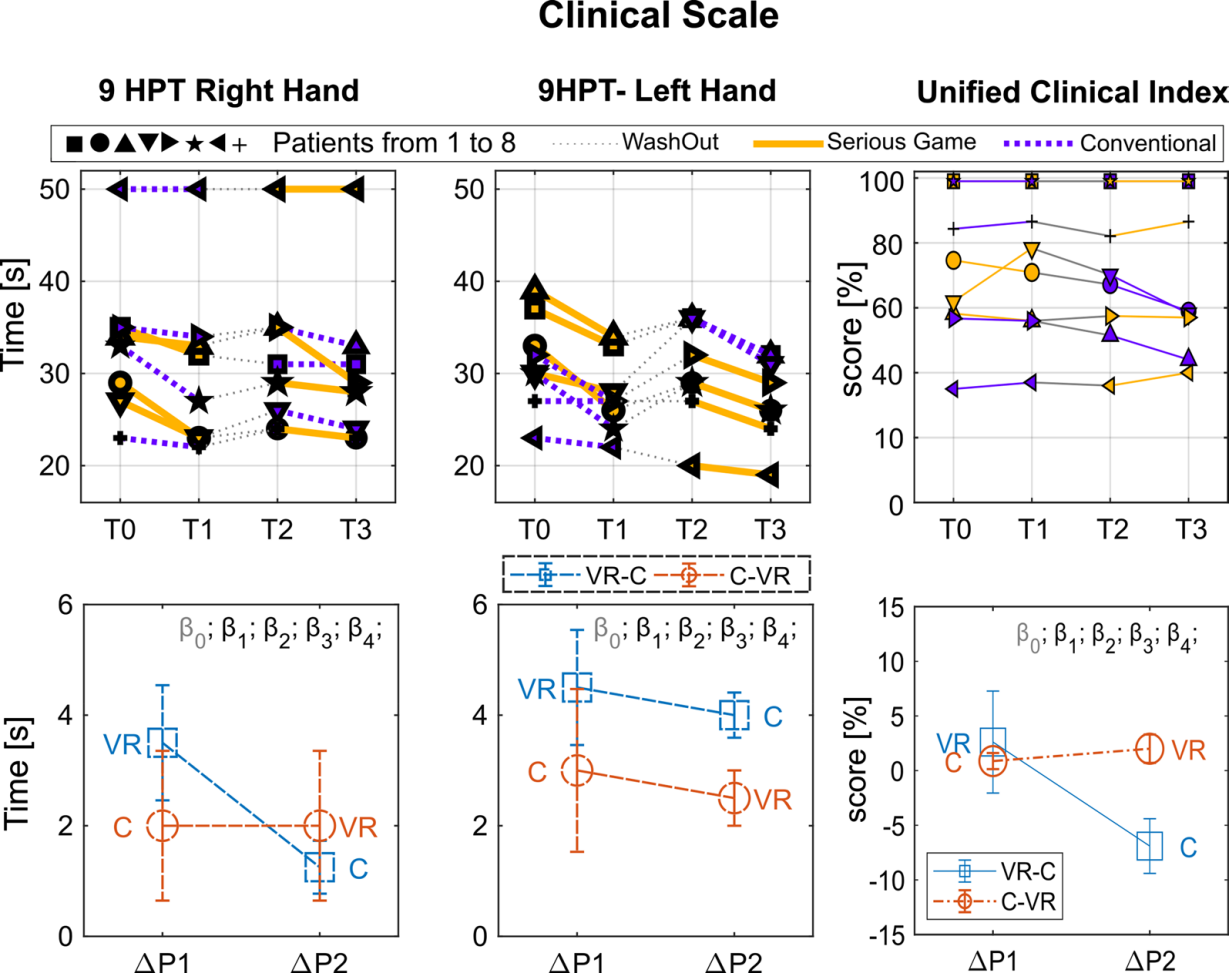
Clinical Scale Results Top graphs: Performance for each subject in the four assessment points: T0, pre-treatment assessment of Period 1; T1, post-treatment assessment of Period 1; T2, pre-treatment assessment of Period 2; T3, post-treatment assessment of Period 2. Period 1, from T0 to T1; Wash out, from T1 to T2; Period 2, from T2 to T3. Each subject is indicated by a different symbol and the kind of treatment he/she underwent in each period is marked by the color of the line (yellow for VR and blue for C). Bottom graphs: Performance changes between post- and pre-treatment measurements ($\Delta P$) averaged over subjects within each period ($\Delta P1$ and $\Delta P2$). In order to improve the readability of the graphs in such a way to have higher values for better performance, $\Delta P$ was multiplied by minus one. Colors distinguish the two groups (experimental sequence). Statistical significances of the $\beta$ LME model parameters are marked through asterisks (see Eq. 1, $\beta _0$ *intercept*, $\beta _1$ *period*, $\beta _2$ *treatment*, $\beta _3$ *baseline*, $\beta _4$ period*treatment)

### Experimental protocol

The study consisted of a total of 16 sessions, 2 sessions per week for 8 weeks divided in two periods by a 4-week wash-out, with a preliminary familiarization and training phase. Before the beginning and after the end of the training phase, we ran the assessment sessions. The familiarization phase had the purpose of practicing the gestures necessary to interact with the VE. It took place once for each subject, at the beginning of the first session. Under the supervision of the therapist, subjects interacted with the VERA gaming scenario for a maximum of 15 min. In this phase a simplified gaming environment was used with a predefined set of gaming levels and parameters. It required specific movement directions (horizontal plane movements, transverse plane movements) and provided to the subject a direct feedback of her/his performance (game scores, time and progress). The training sessions consisted in conventional therapy or VR-assisted rehabilitation, on the basis of the study design shown in Fig. 1. Details of the training sessions and assessment sessions are described in the following paragraphs.

#### Conventional rehabilitation

The focus of what we have called in the present study “conventional” rehabilitation relied on the Cognitive Therapeutic Exercise based on the neurocognitive rehabilitation theory described by the neurologist Carlo Perfetti [25, 26]. According to this theory, sensorimotor recovery is achieved through the activation of those higher cortical functions that underlie learning of new interaction patterns with the surrounding environment. Its effectiveness in neurological patients has been recently demonstrated [27–29].

Movements of the impaired UL were assisted and guided by a trained physiotherapist upon paths or objects with different shape and texture. While performing movements with closed eyes, the participant had both to select and gather relevant tactile and kinaesthetic information to create a visual mental representation of either path or shape perceived, and the corresponding movement, that he/she has to retrieve subsequently in order to recognize the proposed exercise. Every task was proposed as a problem to be solved through cognitive activation of learning strategies. The physiotherapist was constantly tuning the protocol to the participant’s changing level by considering both the results of ongoing exercises and the potential level of development of that specific function. The final goals were to improve reaching, pointing, tracking, grasping and handling of objects. This is the standard rehabilitation approach which is usually provided to the outpatients of the Developmental Neurorehabilitation Unit, Pisa University Hospital.

**Fig. 5:**
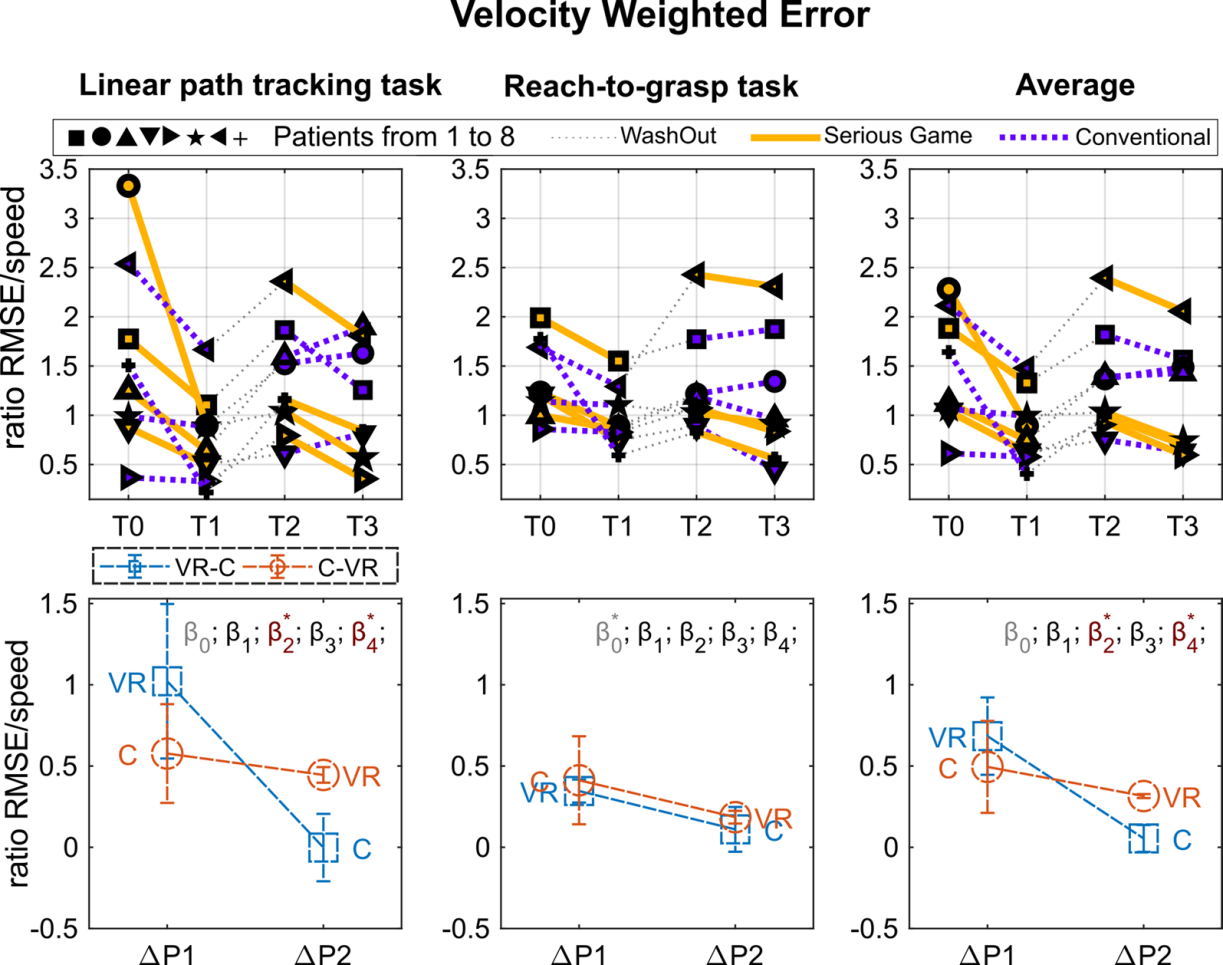
Results of the weighted error metric. Top graphs: Performance for each subject in the four assessment points: T0, pre-treatment assessment of Period 1; T1, post-treatment assessment of Period 1; T2, pre-treatment assessment of Period 2; T3, post-treatment assessment of Period 2. Period 1, from T0 to T1; Wash out, from T1 to T2; Period 2, from T2 to T3. Each subject is indicated by a different symbol and the kind of treatment he/she underwent in each period is marked by the color of the line (yellow for VR and blue for C). Bottom graphs: Performance changes between post- and pre-treatment measurements ($\Delta P$) averaged over subjects within each period ($\Delta P1$ and $\Delta P2$). In order to improve the readability of the graphs in such a way to have higher values for better performance, $\Delta P$ was multiplied by minus one. Colors distinguish the two groups (experimental sequence). Statistical significances of the $\beta$ LME model parameters are marked through asterisks (see Eq. 1, $\beta _0$ *intercept*, $\beta _1$ *period*, $\beta _2$ *treatment*, $\beta _3$ *baseline*, $\beta _4$ period*treatment)

**Fig. 6:**
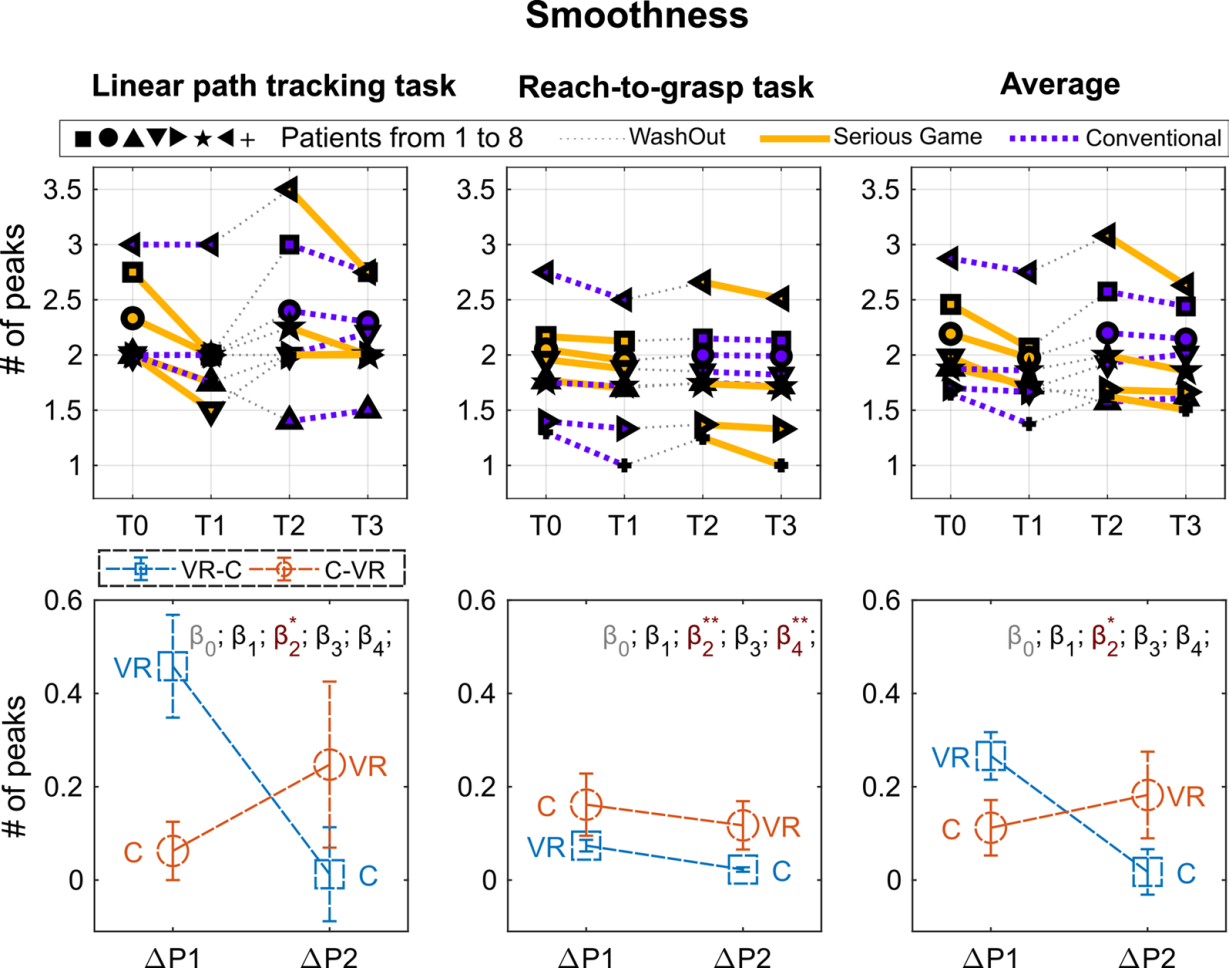
Results of the smoothness metric. Top graphs: Performance for each subject in the four assessment points: T0, pre-treatment assessment of Period 1; T1, post-treatment assessment of Period 1; T2, pre-treatment assessment of Period 2; T3, post-treatment assessment of Period 2. Period 1, from T0 to T1; Wash out, from T1 to T2; Period 2, from T2 to T3. Each subject is indicated by a different symbol and the kind of treatment he/she underwent in each period is marked by the color of the line (yellow for VR and blue for C). Bottom graphs: Performance changes between post- and pre-treatment measurements ($\Delta P$) averaged over subjects within each period ($\Delta P1$ and $\Delta P2$). In order to improve the readability of the graphs in such a way to have higher values for better performance, $\Delta P$ was multiplied by minus one. Colors distinguish the two groups (experimental sequence). Statistical significances of the $\beta$ LME model parameters are marked through asterisks (see Eq. 1, $\beta _0$ *intercept*, $\beta _1$ *period*, $\beta _2$ *treatment*, $\beta _3$ *baseline*, $\beta _4$ period*treatment)

#### VR-assisted rehabilitation

The VR-assisted intervention has been designed to resemble the same motor principles of the conventional treatment: a series of sensorimotor tasks, involving also the cognitive component, proposed within the game setting with hierarchic difficulty. The main feature of the proposed intervention was the patient engagement, achieved by immersion in a virtual gaming scenario: through the visual and auditory channels by means of a Head Mounted Display (HMD, Oculus Rift VK2), and through the sense of touch by means of wearable haptic devices (Fig. 2). An optical tracking system was used (OptiTrackTM V120 Trio, Motive, USA) set at 60 Hz refresh rate of the tracked position. Tracking accuracy within the used workspace was measured by the experimenters and resulted below 0.1 mm. Two serious games have been introduced: “moneybox” game, focused on pincer grasp and reaching tasks with pronation/supination of the hand, and “marble labyrinth” game, focused on pointing and tracking movements requiring both eye-hand and upper limb inter-joint coordination, whose detailed description is provided below.

- Moneybox Game: The player is asked to grasp a golden coin and to insert it into a floating piggy-bank. The player has to collect all the coins that appear on the table paying attention to the position and orientation of the piggy-bank, which rotates clockwise or counterclockwise during the game according to the initial orientation. The position of the objects, the orientation range and tolerances for inserting the coin can be tuned by the therapist through a Graphic User Interface (GUI). The different location and distance of the objects from the body, and the relative orientation of the piggy-bank slot, can be effectively used to train manual reaching, grasping, and forearm pronation/supination.
- Marble Labyrinth Game: a sliding token has to be driven through a labyrinth by dragging it with the fingertip. The therapist can shape the labyrinth and the dragging direction through the GUI, according to the motor capabilities and to the therapeutic purposes of the session. The labyrinth changes as the player increases his/her level and the time to complete the task reduces after each successful progress.

### Assessment

Clinical characteristics of CP and DD children are reported in Table 2.

**Table 2:** Summary of clinical scores at baseline for all the children with developmental disorders

Patient ID	Age	Gender	Diagnosis	GMFCS	MACS	UCI	9-HPT		
							Right	Left	Mean
PA1	14	F	CP	2	1	0.99	45	37	41
PA2	12	M	DD	–	–	0.75	29	33	31
PA3	9	M	DD	–	–	0.57	29	32	30.5
PA4	6	M	DD	–	–	0.58	42	39	40.5
PA5	9	M	DD	–	–	0.62	23	30	26.5
PA6	8	F	CP	1	1	0.99	33	30	31.5
PA7	12	M	CP	1	3	0.35	–	23	23
PA8	11	M	DD	–	–	0.84	23	27	25

We adopted the most commonly used clinical scales for classifying patients abilities in relation to the disease they are affected with: the Gross Motor Function Classification System (GMFCS) [30] and the Manual Ability Classification System (MACS) [31] for CP, and the Zoia’s Protocol (ZP) for DD [32–34]. In addition, ZP scores for DD together with Melbourne Assessment of unilateral upper limb function (MA) scores for CP [35] were considered as secondary outcome measures and, as such, were assessed during the evaluation sessions. In order to compare the results obtained from the two clinical scales (MA, ZP), we elaborated a Unified Clinical Index (UCI) by normalizing the scores of the ZP to match the MA. In particular the ZP consists of a series of tasks which are evaluated with only two possible outcomes: accomplished or not accomplished. The number of accomplished task has been divided by the total number of tasks. Such transform matches the range of possible scores of ZP to the range of scores of MA, so in Table 2 and Fig. 4 a UCI is shown for each participant. Moreover, as a further unifying element beyond the specific characteristics of each diagnostic entity, the Nine Hole Peg Test (9-HPT) [36] was also administered to test for manual dexterity and finger coordination. In fact, 9-HPT is a commonly used test for upper limbs motor abilities in a wide variety of diseases such as stroke, Parkinson’s disease, multiple sclerosis, cerebral palsy and so on [37, 38], which shows a high relationship with both actual upper limb performance in real-life and ADLs.

In addition, kinesiological assessment, as described in detail in Bortone et al. [39], was performed in order to detect any impairment-dependent difference. The kinesiological assessment was carried out in a real setting scenario during two different motor tasks involving the following target actions:

1. Linear path tracking on different directions with respect to the sagittal plane
2. Reach-to-grasp with subsequent pronation/supination of the forearm.

For the linear path tracking (Fig. 3c), the child was seated in front of a desk with the hand positioned at rest close to the body on the sagittal plane. Then, a target was put on the desk in different locations at the same distance from the starting hand position and the child was asked to reach the target by moving the hand along a straight path. For the reach-to-grasp task (Fig. 3d), the child was asked to flip a card protruding from a horizontal support placed at the shoulder height in front of him/her. Each task was performed 6 to 10 consecutive times, during which the hand position and orientation was recorded through an optical tracking system. We analyzed kinematic variables of movements related to reach-to-grasp, with precision pinch and forearm pronation-supination, and linear path tracking in terms of:

- Smoothness of movements: computed as the number of peaks in the speed profile, whose increase correspond to a worse performance;
- Velocity weighted error (V-W Error): in order to balance the effect of the speed on estimated errors [39], we computed the ratio between the movement error (root mean square error from the optimal trajectory) and the mean velocity, lower values correspond to better performance.

### Statistics

In order to decrease the between-subject variation effect, for each subject and for each of the outcome variable the measurements taken at the start of each treatment period were used as baseline values. The change between after-treatment measure and pre-treatement measure within each treatment and period was used as the response variable. A linear mixed-effect (LME) model was applied for analyzing treatment effects, period effects, and carryover effects of the 2 × 2 cross-over study using the following formula in which the carry-over effect is modeled as an interaction effect between the treatment and the period:

1 $$\begin{aligned} y_{ijk}&= \beta _0 + \beta _1 X_{period}+\beta _2 X_{treat}+\beta _3 X_{baseline}+ \\&\quad +\beta _{4} X_{period} X_{treat}+s_{ij}+\epsilon _{ijk}; \end{aligned}$$

where $y_{ijk}$ is the difference between the measurements after and pre treatments (for *i*th sequence, *j*th subject, *k*th period), $s_{ij}$ is the random subject effect term and $\epsilon _{ijk}$ is the error term (both are assumed to be independently and identically distributed). $X_{baseline}$ is the assessment performed at the enrollment; $X_{period}$ and $X_{treat}$ are the two dummy variables taking into account the two periods and the two treatments respectively. In case of significant interaction effect, only the first period is taken into account for determining the difference between treatments. Differences at baseline between the two groups were assessed through a 1-way ANOVA test for each response variable.

## Results

Among the 9 eligible individuals, 8 of them were enrolled in this study and the family agreed to participate (see Fig. 1 for the participants flow). No harms or unintended effects were observed in this RCT study.

The report of the measured performance is divided in the three panels of Figs. 4, 5 and  6, referred to the clinical scale (9-HPT and UFI), to the Velocity Weighted Error, and to the Smoothness metrics respectively. For each panel top graphs show the performance of each subject measured at the four time intervals of the treatment, and bottom graphs the average of the relative increment of the two treatment periods (T1–T0, T3–T2). Results of the kinesiological assessment (Smoothness and Weighted Error) are reported for each of the rehabilitation task (path-tracking, reach-to-grasp, and average).

As explained in “Statistics” section, a single LME model was created and fit for each metric. Significance of the $\beta$ coefficients was reported on the top of each plot. No significant initial differences between groups were found for any of the outcome measurements.

Regarding the clinical scale assessment, an increment of the 9-HPT performance was found in the measured average value after both periods of treatment; in particular for Period 1, the average increment was higher for VR treatment, 3.3/:*s*, than for C treatment, 2.1/:*s*, (9-HPT of the right hand). No significant difference emerged between treatments, while a significant increment with respect to the baseline was measured for the 9-HPT, right hand, of the VR condition in Period 1 (t-test result: t(3) = − 3.36; p = 0.04). No statistically significant difference was found between periods and treatments in the UCI.

Regarding the Linear Path Tracking Task, a Treatment main effect was found for the “Smoothness” index (F(1,11) = 5.0, p = 0.047) and a Treatment * Period interaction effect was found for the “Velocity Weighted Error” index (F(1,11) = 7.037, p = 0.022). It indicates the VR treatment turned out to be more effective than the C treatment in improving the “Smoothness” index regardless of the Period considered (i.e., both in Period 1 and in Period 2).

It should be noted that for the “Velocity Weighted Error” index a Treatment main effect was also found (F(1,11) = 8.7, p = 0.013), however, as suggested by Stevens [40], statistical correctness requires avoiding commenting on main effects in the presence of a “disordinal” interaction, as in the present case.

Regarding the Reach-To-Grasp Task, a Treatment * Period interaction was found only for the “Smoothness” index (F(1,11) = 15.73, p = 0.002). No treatment-induced modifications were demonstrated in this task for the “Velocity Weighted Error” index.

Considering the obtained averaged results, showing a higher increment of performance in Period 1 for both treatments, we conducted a paired t-test between T1 and T0 aggregating the treatments. All the metrics (9-HPT, VW-Error, Smoothness), except the UCI, resulted significantly improved in Period 1 (t-test results: 9-HPT: t(7) = 3.27; p = 0.014, Velocity Weighted Error: t(7) = 3.37; p = 0.012 Smoothness t(7) = 4.07; p = 0.005).

In summary, both treatments showed comparable efficacy in improving the kinesiological outcome in- dices of both target actions (Linear Path Tracking and Reach-To-Grasp Tasks), and showed significant improvement in Period 1. The VR treatment turned out to be more effective than C in improving the “Smoothness” index of the Linear path tracking task regardless of the Period in which it was performed (i.e., both in Period 1 and in Period 2). Period 1 showed in general a more evident increment of performance for both the treatments, with a significant increment of all the metrics (except UCI). The VR treatment resulted also in a significant improvement of the 9-HPT in Period 1 with respect to the baseline. Both treatments showed no apparent efficacy in improving the UCI, and no significant difference was found between the two treatments.

## Discussion

The aim of the present study was to evaluate the efficacy of a 4-week VERA rehabilitation program compared to a more conventional rehabilitation treatment based on physiotherapy in children with neuromotor impairments. Although we hypothesized that VERA would be more effective than conventional rehabilitation, we have shown that the use of VR-assisted therapy was not significantly different from conventional training in terms of improving upper limb functions. Of course the absence of significant difference between treatments can be due to the limited sample size. On the other hand, the sample size was sufficient to highlight a significant improvement in the first period of the two treatments.

Both treatments showed a comparable efficacy in improving the kinesiological indices of either Linear Path Tracking Task or Reach-To-Grasp Task depending on the period in which the treatment was undertaken (Treatment*Period interaction), with a significant improvement in Period 1. Since we performed a $2\times 2$ cross-over study design, we were not able to disentangle how much this interaction depends on a carry-over effect or rather on a sequence effect. We cannot exclude that the chosen wash-out period was too short to allow the complete recovery of the baseline conditions (carry-over effect), which should be achieved to reliably compare the effectiveness of the two treatments. Another possible explanation would be the memory of the previous treatment, which could have played a role in terms of the overall effectiveness of the treatment sequence (sequence effect). Giving the results of the present study, we can argue that VR-assisted therapy is not less effective than conventional therapy and that, therefore, it might be used (a) as an alternative to conventional therapy, for example in a home environment in disadvantaged areas where reaching health facilities would be particularly difficult, or (b) in a complementary manner to conventional therapy, for example providing alternating cycles of conventional therapy in the institutional setting and VR at home, not only to exploit the additive effect without losing effectiveness, but also to optimize time load, care load and management costs for the patient itself, its family and the healthcare system. To further support the similar effect of the two treatments, further experimental results in a larger sample size are required.

Conversely, both treatments showed no apparent efficacy according to the clinical scales (UCI), and limited efficacy for the 9-HPT. Such a mismatch between kinesiological indices of target actions (performed during the Linear Path Tracking Task and the Reach-To-Grasp Task) and clinical performance tests probably lie in a series of concurrent causes without necessarily implying a lack of transfer from the rehabilitation setting. In fact, on one hand, the target actions that were designed for kinesiological analysis could more faithfully reflect the actions carried out during the execution of serious games giving rise to a more direct transfer. Kinesiological indices could be more sensitive than clinical performance tests in detecting subtle improvements in the modalities of execution of the movement. Moreover, the results of clinical performance tests could be burdened by a wide inter-individual variability which, together with the small number of the study group, may have contributed to the failure to achieve statistical significance.

In a previous study conducted on hemiplegic children with CP during reach to grasp [41], it was demonstrated that the head and the trunk are involved and compensatory movements are performed in order to compensate the upper limb and shoulder deficit. Our serious game scenarios have been developed with the possibility to enable the link between the trunk position and a virtual object during the task execution in order to avoid compensation movements. However, in this study, we preferred not to use restraint in order to avoid psychological frustration. Further studies should be conducted integrating this analysis with head and trunk position during the task execution.

The results of the present study show that the VR-assisted therapy with VERA system was overall feasible for introduction in a prolonged rehabilitation trial, in line with previous research [9, 19]. In fact, our findings indicate that VERA improves hand functions in patients with CP/DD at least as much as conventional rehabilitation. Furthermore, it has been reported that no children experienced intervention-related adverse events during VERA and we did not observe any side effects. On the basis of verbal comments by children and observed behaviors, the gaming VR setting was well accepted and seemed to capture interest of the children, especially in the first sessions of the therapy where the use of an HMD display was a novelty for them. With progress of the rehabilitation treatment, children seemed overall acquainted to the VERA system and the initial interest seemed mitigated. It has to be considered that, although immersive VR is not yet widespread in common use, video-games have a far longer story. In the rehabilitation scenario, interests in the use of VR is constantly increasing, and the activities performed with video-based games for UL rehabilitation are considered superior in terms of frequency, repeatability, high motivation, and also in term of innovation for physiotherapists to make treatment entertaining.

However, in the literature, only few RCTs have been found in the rehabilitation of children with CP or DD. In addition, popular most commonly used devices in VR-assisted therapy were commercially available, such as Microsoft Kinect and Nintendo Wii-Fit (see Table 1). These devices fail to detect fine hand and finger movements, which is needed to train dexterity, and existent video game-based applications did not always match with specific therapeutic purposes. Nonetheless, conducted studies reported that VR-assisted therapy was deemed to be more enjoyable than, and preferable to, the conventional therapy because VR-assisted therapy using interactive VR games can provide task-oriented practice, as well as visual and auditory feedback regarding performance and gain, which further motivates and engage players to increase the rehabilitation intensity [42].

Several design factors should be addressed in future research. First, we did not perform power analysis because the participants included to the present study were patients who attended our clinic. So, the sample size is too small for an in-depth analysis, and therefore, any future work should be planned with larger patient groups according to power analysis. Second, the long-term effects of VERA were not evaluated in this study. Third, we think that VR-assisted therapy can increase the motivation of the patients, but we did not evaluate the motivation of patients which could influence the adherence to the treatment.

## Conclusion

The child develops motor, cognitive, emotional and moral behavior through playing and social interaction, which continue throughout life. The variety of games contextualizes and provides adequate motor development, and is fundamental for motor learning. The ability both to customize the modalities of interaction with the virtual environment and, in the same time, to arise motivation in young participants have revealed VR as a potentially important rehabilitation tool in these children [3]. In fact, VR scenarios can offer sensorimotor experiences that are otherwise unfeasible in common therapies [43, 44] and give the opportunity of tailoring therapeutic exercises to meet patients different abilities and needs. Moreover, wearable haptic devices introduce an added value since they provide the opportunity to enrich sensory feedback (and thus to foster neuroplasticity) by maintaining the ease of set up and, at the same time, without hindering fingers movements.

Considering the promising results regarding the proof of concept trial of the proposed VR rehabilitation previously obtained [1, 39], a phase-2 pilot clinical trial was then carried on. Results have quantitatively shown that the serious games coupled with wearable haptic devices can be designed as an effective treatment. Given the limits of the limited sample size, we argue that VERA is a feasible and effective alternative training for children with neuromotor impairments. In addition, no adverse effects of VERA were observed.

## Perspectives

We believe that further work should be conducted in this area. The inclusion of diplegic participants could provide the opportunity to make a deeper investigation including the comparison of the effects between diplegic and hemiplegic children. In addition, further longitudinal studies should be conducted in order to assess the residual effects of the treatment in these patients. We will also consider the inclusion of psychological assessment of the children before and after the treatment, in order to measure the effective level of engagement and motivation we would be able to arise. Finally, further crucial data may be given by electromyography, which may provide addition information on effects of upper limb muscle activity on kinematic parameters over time.

## Data Availability

The datasets used and analysed during the current study are available from the corresponding author on reasonable request.

## Abbreviations

VERA: Virtual Environments and weaRable hAptic devices
SG: Serious Game
RCT: Ranzomized controlled trial
UL: Upper limb
UE: Upper extremity
ABI: Acquired brain injury
JIA: Juvenile idiopathic arthritis
BPBI: Brachial plexus birth injury
CP: Cerebral Palsy
DCD: Developmental coordination disorder
DD: Developmental dyspraxia
ses: Sessions
wks: Weeks
VR: Virtual reality
LMC: Leap motion controller
PT: Physical therapy
OT: Occupational therapy
CIMT: Constraint-induced movement therapy
CG: Computer Games
NTT: Neurodevelopmental therapy
CONV: Conventional therapy
+: In conjunction
-: Subsequent
9-HPT: Nine Hole Peg Test
UCI: Unified Clinical Index
GMFCS: Gross Motor Function Classification System
MACS: Manual Ability Classification System
MA: Melbourne Assessment
ZP: Zoia’s Protocol
V-W Error: Velocity Weighted Error
